# The Immunosuppressant Protosappanin A Diminished Recipient T Cell Migration into Allograft via Inhibition of IP-10 in Rat Heart Transplant

**DOI:** 10.1371/journal.pone.0096138

**Published:** 2014-05-05

**Authors:** Maomao Zhang, Jieqiong He, Jingbo Hou, Jian Wu, Meng Sun, Jinjin Cui, Jiangtian Tian, Miaomiao Jiang, Bo Yu

**Affiliations:** 1 The Key Laboratory of Myocardial Ischemia, Chinese Ministry of Education, Harbin, Heilongjiang Province, China; 2 Department of Cardiology, Second Affiliated Hospital of Harbin Medical University, Harbin, Heilongjiang Province, China; Université Libre de Bruxelles, Belgium

## Abstract

The immunosuppressant Protosappanin A (PrA), isolated from the medicinal herb, promotes cardiac allograft survival, diminishes inflammatory cell infiltration, and inhibits interferon γ-induced protein 10 kDa (IP-10) mRNA expression in rats cardiac grafts. Binding of the chemokine IP-10 to its cognate receptor, CXCR3, plays crucial roles in allograft immunity, especially by mediating the recruitment of effector T cells to allografted tissues. In this study, we attempted to determine whether PrA-mediated inhibition of IP-10 contributes to the effect of reduced T cell infiltration into cardiac allograft within a rat model. Administration of PrA (25 mg/kg daily) via oral gavage following heart transplantation significantly reduced the increase of IP-10 mRNA level in allograft and prevented IP-10 secretion by peripheral blood mononuclear cells (PBMC) isolated from recipient rats seven days posttransplantation. Furthermore, *in vitro* experiments demonstrated that PrA addition to control PBMC prevented IP-10 secretion. Chemotactic migration assays were utilized to evaluate recipient T cell migration towards PBMC supernatant. PrA administration impaired PBMC supernatant-induced T cell migration. Additional *in vitro* experiments revealed that PrA slightly reduced naïve T cell migration towards chemokines. The presence of IP-10 in PBMC supernatant prevented PrA from reducing T cell migration in PrA-treated recipients. Neither CXCR3 chemokine ligand Mig nor non-CXCR3 chemokine ligand SDF-1 had any effect on T cell migration in PrA-treated recipients. The addition of anti-CXCR3 antibody restored PrA-mediated inhibition of T cell migration. Immunofluorescence microscopy showed that IP-10 was expressed mainly in CD68 positive infiltrating monocytes. Furthermore, PrA consistently reduced CXCR3^+^T cell infiltration into cardiac allografts. The reduced intensity of CXCR3 staining in PrA-treated allografts contributed to the previously depressed naïve T cell migrating activity induced by PrA. Collectively, these data indicate that PrA inhibition of IP-10 activity reduced recipient T cell migration and infiltration of cardiac allografts, thus partially explaining the immunosuppressive effect of PrA.

## Introduction

The Chinese herb *Caesalpinia sappan* L. shows biological activities and provides therapeutic potential for some diseases, ranging from autoimmune disease to cancer [1]–[5]. Recent research efforts have aimed to isolate and identify the bioactive components of this Chinese herb in order to generate more robust drug therapies and gain a better understanding of the molecular mechanisms underlying its therapeutic effects. Thus far, an ethanol isolated extract from *Caesalpinia sappan* L., protosappanin A (PrA), was found to have remarkable anti-rejection activity [6]. In particular, PrA treatment of a rat heart transplant prolonged graft survival, alleviated pathologic damage, and reduced infiltration of mononuclear cells into the graft [7]. Molecular studies have suggested that the mechanisms by which PrA protects cardiac allografts from acute rejection may involve the suppression of NF-κB activation and the reduced expression of interferon γ-induced protein 10 kDa (IP-10) [7]. However, the precise underlying immunosuppressive mechanism of PrA remains to be fully elucidated.

IP-10, which is secreted by immune cells and is a potent chemoattractant for T cells, dendritic cells and macrophages, interacts with its receptor CXCR3 to regulates chemotaxis during inflammatory or immune responses [8]–[11]. In particular, IP-10 mediates chemotaxis and infiltration of mononuclear cells during the inflammatory response, which is associated with allograft rejection after transplantation [9], [12]–[15]. The interaction of IP-10 with CXCR3 induces T cells recruitment to inflammatory sites [16]. The cardiac allografts from IP10^-/-^ donors have been shown to promote graft survival after heart transplantation [17]. Consistent with a role in immune responses during allograft transplantation, up-regulation of the circulating IP-10 may be used as a predictive marker or a risk factor for allograft rejection [15], [18].

We have previously demonstrated that PrA significantly reduced mRNA expression of IP-10 within the cardiac graft [7]. In the study, we tried to evaluate the effect of PrA addition on IP-10 expression and T cell migration into allografts. Our results suggest that PrA inhibited IP-10 expression in the graft heart. Accordingly, *in vitro* application of PrA to peripheral blood mononuclear cells (PBMC) reduced IP-10 secretion, prevented T cell migration, and identified a potential mechanism for PrA-mediated inhibition of CXCR3^+^T cell infiltration into cardiac allografts.

## Materials and Methods

### Drug preparation

PrA was extracted from the heartwood of *Caesalpinia sappan* L.and identified by wave spectrum with a >98% purity as described previously [6], [7], [19]. PrA was then dissolved with sterile distilled water at the appropriate concentrations. To rule out possible endotoxin contamination of PrA samples, we performed an endotoxin content assay (LAL assay) and comfirmed the endotoxin levels with less than 5 EU/mg compound.

### Heart transplantation

We performed ectopic peritoneal heart transplantations from DA to Lewis rats using previously published method [20]. Male Lewis (recipient; grade specific pathogen-free; 200–250 g) and DA rats (donor; grade specific pathogen-free; 180–220 g; the Experimental Animal Center of Beijing, China) were used in heart transplantation. The cardiac allograft function was monitored daily through abdominal palpation for nine days posttransplantation. The cardiac allograft was excluded from the experiment when palpated with no contraction, considered as acutely rejected.

After heart transplantation, the recipient rats were divided randomly into two groups and given the following treatments from day two to seven after operation: 1) sterile distilled water (control), or 2) daily administraton of 25 mg/kg PrA by oral gavage. In the majority of the experiments, eight animals per group were used, unless otherwise indicated. Seven days post-operation, blood samples and graft hearts were harvested according to the published methods [7].The animals were maintained in accordance with laboratory animal care principles and use guidelines, and the experiment was approved by the Animal Ethics Committee at the Harbin Medical University.

### Quantitative RT-PCR (qRT-PCR)

qRT-PCR was performed using previously published protocols [7], [21]. Briefly, snap-frozen graft pieces were crushed and total RNA was harvested from cardiac allograft by Trizol Reagent (Invitrogen, CA, USA). Total RNA was reverse transcribed into cDNA (Promega, Madison, WI, USA), and the genes of interest were amplified using SYBR Premix Ex Taq II and the following protocol: 94°C for 5 min, 30 cycles of 94°C for 30 s, 50°C for 30 s, and 68°C for 30 s. The differential expression of several genes was evaluated and GAPDH as an internal control was included. IP-10–forward, 5′-TTATTGAAAGCGGTGAGCCAAAG-3′ and reverse, 5′-GGACAGTTAGGACTAGCCGCAC-3′; Mig–forward, 5′- AATCCCTCAAAGACCTCAAACAGT-3′ and reverse, 5′- GCAGGTTTGATCTCC GTTCTTC-3′; ITAC–forward, 5′-GCAGAATTCTGCAGCGGCTGCTGAGATGAACAG-3′ and reverse, 5′- GGACCTTCTAGAAAGTTCTGCAGC-3′; RANTES–forward, 5′- ACCATGAAG GTC TCCGCG -3′ and reverse, 5′- TTCAGGTTCAAGGACTCTCCA -3′; GAPDH–forward, 5′-TTCATTGACCTCAACTAC-3′ and reverse, 5′-AGACTCCACGACATACTC-3′. The GAPDH primers used in this study were previously published [7], [21].

### PBMC isolation from recipient rats

PBMC were isolated from blood samples of the recipient rats as previously reported [6]. Cells were washed and resuspended in RPMI 1640 medium (Hyclone, Logan, USA) supplemented with 10% FCS (Hyclone, Logan, UT, USA). The PBMCs were cultured with the concentration of 2×10^6^ cells/ml for 4 h (37°C, 5% CO_2_), followed by collection of the culture supernatant for further evaluation. Prior to use, cells conditioned with different concentrations of PrA (0, 5, and 20 nM) were incubated for 72 h.

### Enzyme-linked immunosorbent assay (ELISA)

PBMCs isolated from either non PrA-treated or PrA treated recipient rats on day seven post transplantation were cultured at the concentration of 2×10^6^ cells/mL for 4 h. In the another experiment, PBMCs isolated from control recipient rats on day four post transplantation were cultured with different doses of PrA for 72 h. According to the manufacturer's instructions, IP-10 levels in the PBMC supernatant were measured by ELISA kits (USCN Life Science, Houston, USA).

### Cell viability by MTT assay

Cell viability of PBMCs following PrA treatment with different doses (0, 5, 20, and 40 nM for 48 h) was determined using previously published protocol for the MTT assay [19].

### Chemotaxis assay

Chemotactic activity was measured by a chemotactic migration assay in transwell cell culture chambers with polycarbonate membranes (5 µm diameter pore size, Costar, Cambridge, MA, USA). The recipient spleen T cells were harvested using the following procedure. After anesthetization spleens were removed aseptically, cut, and passed through a wire mesh to obtain a single cell suspension. Red blood cells were lysed by addition of red blood cell lysis buffer (Beyotime, Haimen, China), and T cells were purified from splenocytes by nylon wool filtration. T cells purity was assessed with an anti-CD3 fluorescent marker by flow cytometry (>95%). T cells were cultured in RPMI 1640 medium (Hyclone, Logan, UT, USA) with 10% FCS (Hyclone, Logan, UT, USA). T cells (100 µl at 1×10^6^ cells/ml) were harvested and then added to the upper chamber. Meanwhile, supernatants of PBMC cultures from recipient rats with or without PrA administration were collected and added to the lower chamber. The migration of T cells to supernatant of PBMCs was assessed under four different conditions: 1) T cells alone; 2) cell-free PBMC supernatant; 3) T cells co-cultured with autologous PBMC supernatant; and 4) crossover culture consisting of T cells from PrA group rats co-cultured with the PBMC supernatant in control group, or T cells from control group rats were co-cultured with PBMC supernatant in PrA group. Chambers were incubated for 4 h at 37°C. Afterwards, chemoattracted T cells were removed from the lower compartment and those positive for CD3 staining were counted with flow cytometry. The ratio of migrating cells was calculated by dividing the cells number in the lower chamber by the total input cells count added to the upper chamber prior to migration [22]. The mechanism of PrA-mediated reduction in T cell migration was evaluated by administrating recipient T cells with PrA-containing PBMC supernatant supplemented with either rrIP-10 (50 ng/ml), rmMig (100 ng/ml) or rrSDF-1 (100 ng/ml, PeproTech, NJ, USA). Moreover T cells were treated with anti-CXCR3 Ab or IgG isotype control Ab (20 µg/ml; Santa Cruz Biotechnology, CA, USA). Transmigration was then assessed after 1 h.

### Immunocytochemistry and confocal microscopy

Seven days posttransplantation, heart allografts were dissected and sectioned for staining of CXCR3, TCR and CD68 by immunocytochemistry as previously published [7]. The primary Abs included rabbit anti-CXCR3 Ab (1∶50, Santa Cruz Biotechnology, USA), mouse anti-T cell receptor (TCR, surface marker of T cell) Ab (1∶50, Santa Cruz Biotechnology, USA), rabbit anti-CD68 Ab (1∶200, Abcam, Cambridge, UK) and mouse anti-IP10 Ab (1∶100, Biorbyt, Cambridge, UK). The secondary Abs included FITC-conjugated goat anti-rabbit Ab (1∶200, Santa Cruz Biotechnology, CA, USA) and TMRITC-conjugated goat anti-mouse Ab (1∶200, Santa Cruz Biotechnology, CA, USA) for 1 h at 37°C. Nuclei were counterstained with DAPI (Sigma, St. Louis, MO, USA).

Within three samples selected from each group, one from series of sections was randomly selected for analysis. Relative quantitative analysis was conducted to determine percentage of CXCR3^+^TCR^+^T cells within infiltrating cells (100 cells evaluated) in cardiac allograft enumerated from ten randomly selected microscopy fields for each section.

### Statistical analysis

Results were expressed as means ± standard errors (SE). The distributed data were compared by one-way ANOVA. Statistically significant differences were defined as *p*<0.05.

## Results

### PrA blocked IP-10 expression in cardiac graft of recipient rats

PrA has been shown to promote allograft survival and reduce the infiltration of mononuclear cells in allografts [7]. Furthermore, several groups have demonstrated the importance of IP-10-mediated recruitment of T cells to allografts and identified the correlation between the development of acute rejection and the increased allograft expression of IP-10 [9], [23], [24]. Therefore, in this study we evaluated whether PrA inhibition of IP-10 expression results in reduced T cell infiltration into heart allografts.

**Figure 1:**
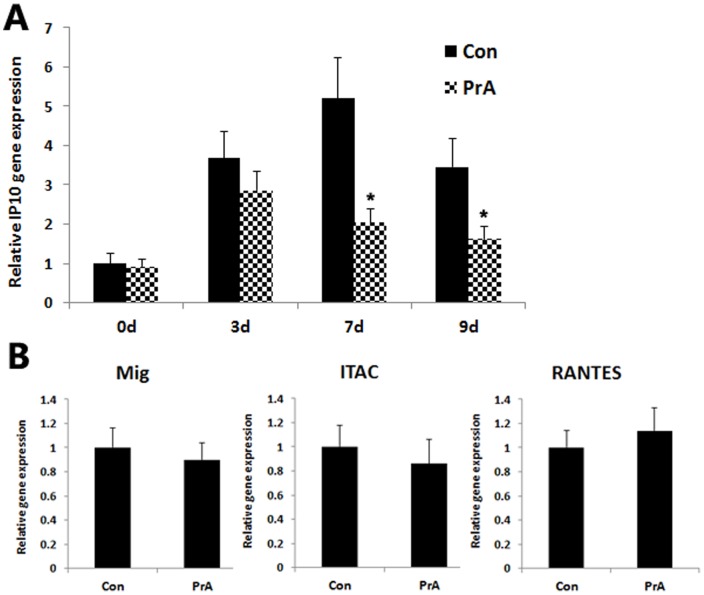
PrA depressed IP-10 mRNA level in allografts. (A) The effect of PrA administration on IP-10 mRNA expression in heart allografts of recipient rats was assessed by relative qRT-PCR on day 0, 3, 7, and 9 posttransplantation. The IP-10 mRNA level decreased with PrA addition (n = 8) as compared to controls (n = 8) on day seven and nine. (B) The gene expression of Mig, ITAC and RANTES was assessed in cardiac allografts on day 7 posttransplantation. However, no significant difference between control (n = 8) and PrA treated group (n = 8) was detected. * indicates *p*<0.05 when comparing treatment to control.

First, the IP-10 mRNA expression in cardiac grafts of recipient rats was assessed by relative qRT-PCR. Consistent with previous work, IP-10 expression in recipient heart allografts dramatically increased, which peaked on day seven post-heart transplantation. PrA administration significantly reduced IP-10 mRNA expression as compared to control heart allografts at seven days posttransplantation (Fig. 1, *p*<0.05). Furthermore, the mRNA expression of other CXCR3 ligands, Mig and ITAC, as well as CCR5 ligands RANTES, was measured in the cardiac allografts on day seven. However, PrA showed no significant effect on the expression of these genes. This data is consistent with previously published microarray data conducted with cardiac allografts [7].

### PrA reduce IP-10 secretion from PBMCs *in vivo* and *in vitro*

**Figure 2:**
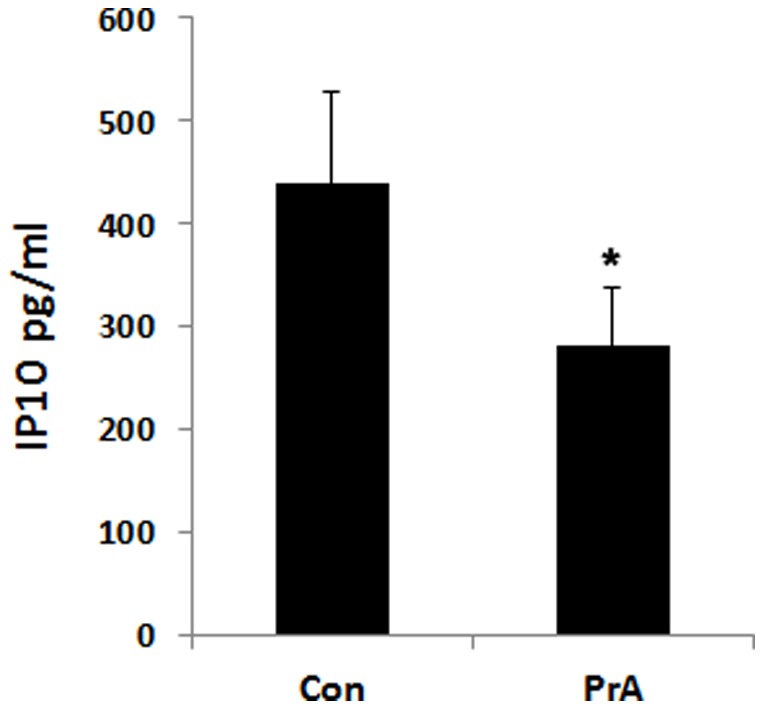
PrA treatment reduced IP-10 secretion from PBMCs in recipient rats. Control and PrA-treated PBMCs from recipient rats (n = 8) were separately isolated on day seven posttransplantation and cultured at a concentration of 2×10^6^ cells/ml for 4 h. Culture supernatant was harvested and analyzed for the IP-10 secretion by ELISA. * indicates *p*<0.05 when comparing treatment to control.

Since mononuclear cells such as monocytes predominantly secrete IP-10 during inflammation and allograft rejection, the effect of PrA on PBMC IP-10 secretion was analyzed. Seven days posttransplantation, recipient rat PBMCs were isolated and analyzed for IP-10 secretion into culture supernatant. PBMCs from PrA-treated recipient rats demonstrated a significant reduction in IP-10 production as compared to control recipient rats (Fig. 2).

**Figure 3:**
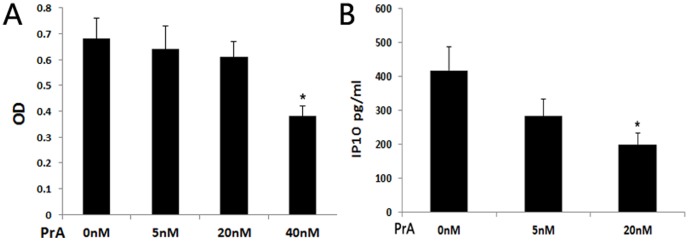
PrA conditioning reduced PBMC IP-10 secretion *in vitro*. PBMCs isolated from control recipient rats on day four were cultured with different doses of PrA for 72(n = 3 per group). (A) PBMC viability was measured by MTT assay. The highest dose induced a significant decrease in viable cell count, therefore, only 5 nM and 20 nM concentrations were used in subsequent experiments. (B) Culture supernatants of PBMC with or without PrA condition were harvested and analyzed for the production of IP-10 by ELISA. * indicates *p*<0.05 when comparing treatment condition to control.

*In vitro* experiments were performed to further assess the PrA inhibition of IP-10 secretion from PBMCs. PBMCs were isolated from recipient rats on day four posttransplantation and treated with indicated doses of PrA for 72 h. The non-toxic dose of PrA was determined by MTT *in vitro*. PrA addition at 40 nM depressed the viability of PBMCs compared with control cultures (no PrA), but lower doses did not (Fig. 3A). Therefore, we used 5 nM and 20 nM doses of PrA for subsequently examining the effect of PrA on PBMCs. ELISA was used to quantify IP-10 secretion in harvested cell culture supernatants. PrA-exposed PBMCs secreted significantly less IP-10 than non-treated cells (*p*<0.05), and PrA addition demonstrated a dose dependent reduction in IP-10 production (Fig. 3B).

### PrA reduced the migration of recipient T cells towards the supernatant of PBMCs

**Figure 4:**
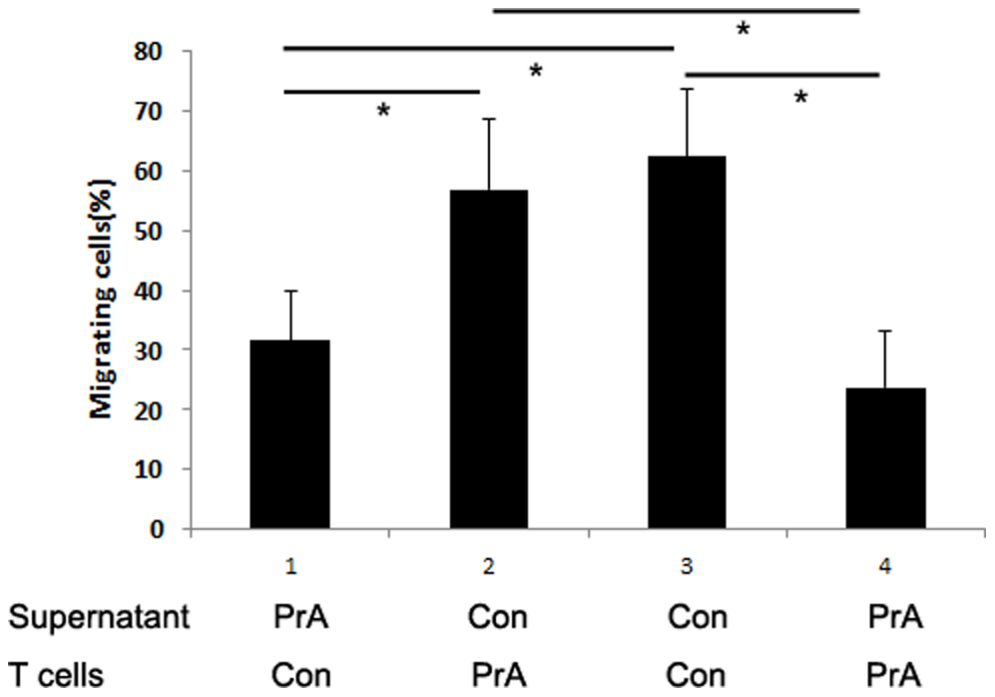
PrA inhibited recipient T cell migration towards PBMC supernatant. PBMC and spleen T cells were isolated from recipient control and PrA-treated rats on day seven posttransplantation. The migration of T cells toward PBMC supernatant was tested using a chemotactic migration assay. In the presence of PBMC supernatant from control recipients, the migration of T cells from PrA-treated recipients was not impaired compared with those from control recipients. However, in the presence of PBMC supernatant from PrA-treated recipients, the migration of T cells was significantly impaired when cells were harvested from either PrA-treated or control recipients. * indicates *p*<0.05, and bars indicate comparators.

Because reduced IP-10 secretion from PBMCs results in altered T cell migration [24], the effect of PrA addition on recipient T cell migration towards PBMC supernatant was assessed utilizing the chemotactic migration assay. The PBMCs and spleen T cells from recipient rats were isolated and cultured on day seven posttransplantation. The migration of T cells, harvested from either PrA-treated recipients or the control recipient rats, was impaired in the presence of PrA-treated PBMC supernatant as compared to control supernatant (Fig. 4). In contrast, in the presence of no-PrA control PBMC supernatant, there was no significant difference in migrating activity between T cells of PrA-treated recipients and those of non PrA-treated recipient rats (Fig. 4). These experiments revealed that PrA inhibited T cells migration towards PBMC supernatant while not overtly altering the capacity of T cell migration.

**Figure 5:**
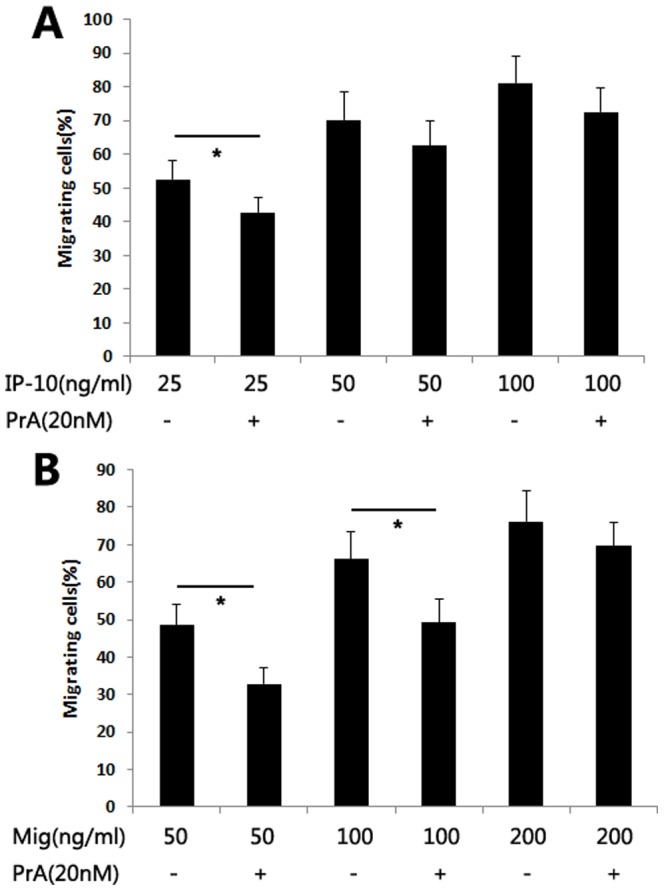
The migrating capacity of naïve T cells was slightly affected by PrA. The naïve T cells isolated from rat spleen were cultured with or without PrA (20 nM) for 72 h. A chemotactic migration assay was used to assess the migration of naïve T cells in the presence or absence of PrA towards different doses of either recombinant IP-10 or Mig. (A) PrA-conditioned T cells exhibited decreased migration in presence of 25 ng/mL of IP-10, but not at 50 ng/mL or 100 ng/mL, compared with those of non-condition T cells. (B) The migration of PrA-conditioned T cells was decreased at 50 ng/mL and 100 ng/mL of Mig, but not at 200 ng/mL, as compared with those of non-conditioned T cells. The result suggests that the capacity of T cell migration was partially affected by PrA *in vitro*. * indicates *p*<0.05, and bars indicate comparators.

To clarify the role of PrA-mediated inhibition of T cell migration *in vivo*, different doses of recombinant IP-10 and Mig were used to test the effect of PrA on the migration of T cells from naïve rats *in vitro*. The migration of PrA-conditioned T cells decreased at low Mig and IP-10 concentrations (25 ng/mL and 50 ng/mL, respectively) compared with those of non-condition T cells. No significant effect of PrA on T cell migration was found at high levels of either IP-10 or Mig (100 ng/mL and 200 ng/mL, respectively, Fig. 5). Although these results show that T cell migration was partially affected by PrA *in vitro*, the effect of PrA on the recipient T cell migration *in vivo* is still not clear.

### PrA inhibition of IP-10 reduced T cell migration *in vitro* and in allografts

**Figure 6:**
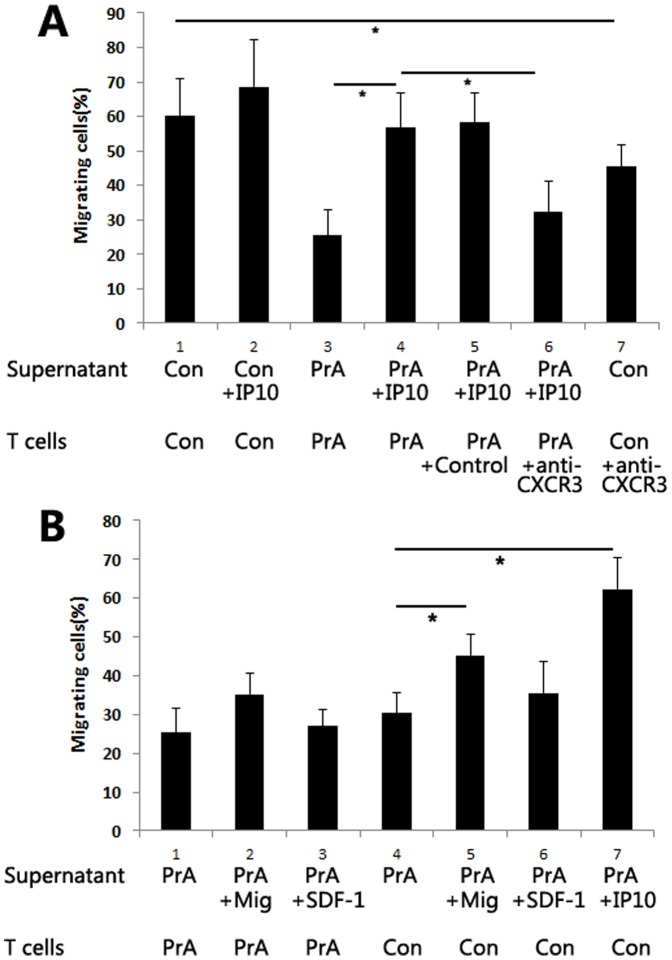
PrA reduced T cell migration to PBMC supernatant through inhibition of IP-10-CXCR3 receptor interaction. IP-10 (50 ng/ml), Mig (100 ng/ml), or SDF-1 (100 ng/ml) was added to the PBMC supernatant. T cells were incubated with the isotype control Ab (20 µg/ml) or anti-CXCR3 Ab (20 µg/ml) prior to chemotactic migration assays (n = 3 in each group). (A) IP-10 addition to the supernatant of PrA-exposed PBMCs improved the migration of PrA-treated recipients T cells. Furthermore, addition of anti-CXCR3 Ab restored PrA inhibition of IP-10-induced T cell migration, but isotype Ab did not have the same effect. (B) The addition of Mig and SDF-1 did not significantly increase the migration of PrA-treated recipients T cells. However the addition of Mig, but not SDF-1 addition increased the migration of non PrA-treated recipients T cells. *indicates *p*<0.05, and bars indicate comparators.

IP-10 functions as a lymphocyte attractant and mediates infiltration of activated T cells expressing CXCR3 into inflamed tissues or allografts [9], [14].We sought to determine the effect of PrA on IP-10-induced T cell migration. IP-10 addition to the supernatant of PrA-exposed PBMCs significantly increased the T cell migration. The addition of anti-CXCR3 Ab depressed the migration of non-PrA-treated recipients T cells towards non PrA-exposed PBMC supernatants, which suggests that anti-CXCR3 Ab is neutralizing antibody (Fig. 6A). Subsequent anti-CXCR3 Ab addition to IP-10 and PrA-treated T cells restored PrA-mediated inhibition of T cell migration (Fig. 6A), suggesting that PrA reduced the T cells migration through IP-10 inhibition. However, addition of either Mig or SDF-1 did not have any effect on the migration of PrA-conditioned recipient T cells. Meanwhile, addition of Mig to the supernatant of PrA-exposed PBMC restored the migration of non-PrA conditioned recipient T cells, but not SDF-1 (Fig. 6B). SDF-1 had no effect on T cell migration, suggesting that PrA mediates its effects via the CXCR3/chemokine ligand interaction and that IP-10 is more sensitive and specific chemokine by which PrA mediates its effects.

**Figure 7:**
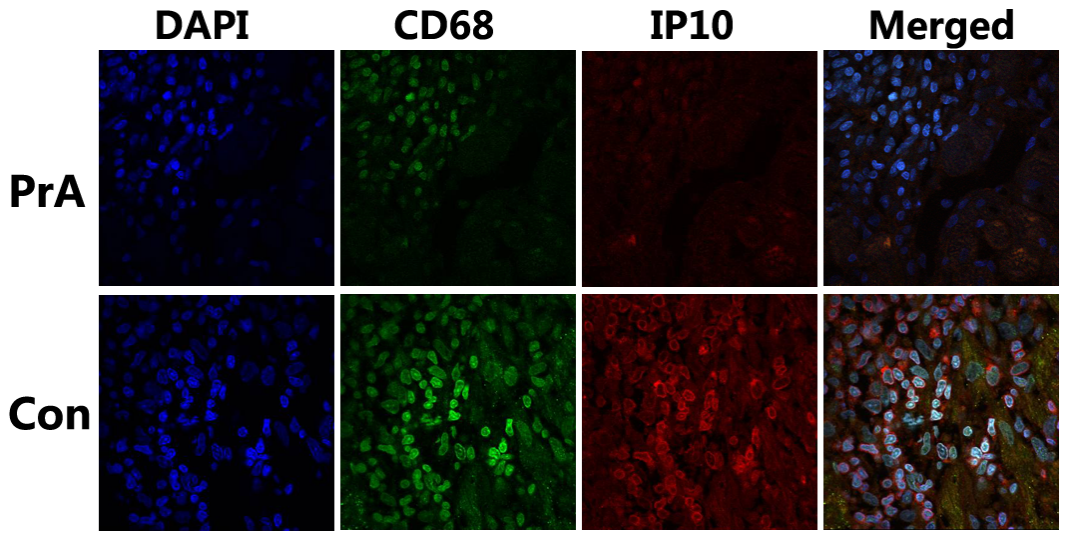
PrA inhibited IP-10 expression in infiltrating monocytes of cardiac allografts. Immunofluorescence staining of heart allograft sections stained with Abs to CD68 (FITC, green) and IP-10 (TMRITC, red). Nuclei were counterstained with DAPI (blue). The IP-10 expression in CD68 positive infiltrating monocytes was reduced after PrA treatment. The results are representative of three independent experiments (n = 3).

IP-10 is expressed not only by mononuclear cells, but also by vascular and stromal cells. Immunohistochemistry results of the cardiac allografts showed that IP-10 mainly expressed in infiltrating mononuclear cells, especially in CD68 positive monocytes in cardiac allografts (Fig. 7). Moreover PrA reduced the IP-10 expression in CD68 positive monocytes (Fig. 7), further supporting the inhibitory effect of PrA on IP-10 expression.

**Figure 8:**
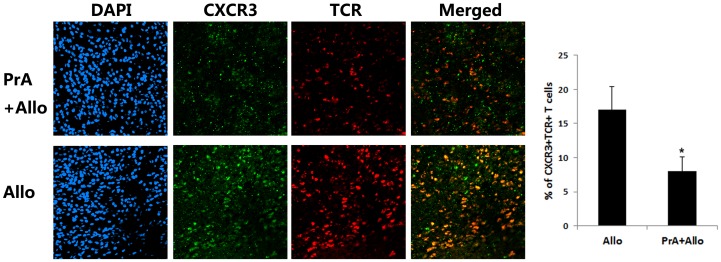
PrA prevented CXCR3^+^T cell infiltration into allografts. (A) The heart allograft was harvested on day seven post heart transplantation, sectioned, and stained for TCR (TMRITC, red) and CXCR3 (FITC, green). The extent of colocalization (yellow) of TCR (T cell marker) and CXCR3 indicating CXCR3^+^ T cell infiltration into allografts was significantly reduced after PrA administration. The results are representative of three independent experiments. (B) The relative quantitative analysis to determine the percentage of CXCR3^+^TCR^+^ cells within infiltrating cells of allografts further showed that PrA treatment inhibited the CXCR3^+^ T cell infiltration into allografts. *indicates *p*<0.05 when comparing treatment to control.

The IP-10 recruitment of antigen specific T cells into allograft appears to partially account for the mononuclear infiltration into allografts. To confirm these data, the *in vivo* PrA effect on IP-10-mediated T cell migration into rat allografts was evaluated. Immunofluorescence microscopy and relative quantitative analysis demonstrated that infiltration of CXCR3^+^TCR^+^ cells into cardiac allografts was significantly decreased in the PrA-treated group as compared with the control group (Fig. 8), suggesting that PrA reduced CXCR3^+^T cell infiltration of allograft hearts *in vivo*. Meanwhile, the intensity of anti-CXCR3 staining in the PrA-treated allografts was dramatically reduced, which might have contributed to the inhibition of T cell migration by PrA. Collectively, these results suggest that PrA blockade of effector T cell infiltration into allografts results from IP-10/CXCR3 axis inhibition.

## Discussion

In previous work, we have demonstrated the immunosuppressive effect of PrA on cardiac allografts. Subsequent reports have demonstrated that PrA inhibits NF-κB activity and IP-10 gene expression, which might be partially responsible for the beneficial PrA-induced immunosuppression [7]. Chemokines play an important role in recruiting effector T cells to allografts, especially IP-10 and its receptor CXCR3, which have been shown to play a crucial role in heart allografts rejection [25]. Therefore, in this study we attempted to further elucidate the mechanism of PrA inhibition of IP-10.

In heart-transplanted recipient rats, PrA inhibited IP-10 production in allografts and reduced CXCR3^+^T cell infiltration into heart allografts. It has been reported that the alloantigen-specific T cells in allografts mainly contribute to the immune response of acute allograft rejection, especially its infiltration and effector functions [26], [27]. Chemokines function as chemoattractants providing signals mediating selective recruitment of mononuclear cells, which may be critical in the acute rejection. IP-10 acts as a potent chemoattractant for the T cells recruitment during the immune or inflammatory responses. The chemokines, especially IP-10, play a pivotal role in the T cells recruitment into allografts [18], [28], [29]. Some studies have found the association between IP-10 expression in graft and acute rejection of cardiac or other organ transplantation [24], [26], [30]. Furthermore, IP-10 neutralization within the donor heart has been shown to prolong allograft survival[17], [31]. These reports are consistent with our results, which suggest that the reduced IP-10 production in the presence of PrA might be partially responsible for the diminished infiltration of T cells into allografts. Therefore, the mechanism of PrA immunosuppression may be the reduction in IP-10 expression and activation of its receptor CXCR3. CXCR3 has multiple ligands, and several chemokines have been implicated in recruitment of T cells into allografts. Therefore, the gene expression of additional chemokines (the other CXCR3 ligands, Mig and ITAC as well as CCR5 ligand RANTES) in cardiac allografts in both the control and PrA-treated group was assessed. The results further suggest that IP-10 might be the specific chemokine by which PrA mediates its effects.

Furthermore, our results demonstrate a decrease in IP-10 secretion by PBMCs in the presence of PrA. IP-10 inhibition by PrA was mainly observed with infiltrating monocytes in allografts and resulted in the reduction of CXCR3^+^T cell allograft infiltration. The decrease in the intensity of anti-CXCR3 staining in the PrA-treated grafts suggests that CXCR3 expression was reduced in the infiltriting cells within allografts, which might be responsible for the slightly depressed migration capacity of T cells induced by PrA. In addition, the chemotaxis assay suggests that reduction in T cell migration of the PrA-treated recipient towards PBMC supernatant by PrA was only restored by IP-10 addition. Neither Mig nor SDF-1 had any restorative effect on T cell migration. The addition of Mig to PBMC supernatant restored T cell migration towards PrA-conditioned PBMC supernatant only in the non PrA-treated recipient. These data further support our hypothesis that PrA inhibition of IP-10 is mainly responsible for reduced recipient T cell migration and infiltration of rat cardiac allografts. Results of this study are consistent with the emerging opinions that although IP-10 and the related chemokines, Mig and i-TAC, interact with the same receptor, CXCR3, they all exhibit unique expression levels and functional patterns *in vivo* [32]-[34]. Within the three CXCR3 ligands, IP-10 is unique in that its promoter has two functional NF-kB binding sites, whereas the Mig and i-TAC promoters have none [35], [36]. Current research needs to include other CXCR3 ligands using targeted deletion of these ligands to address the potential roles of these chemokines in the mechanism of PrA. Furthermore, the mechanism by which PrA inhibits IP-10 expression and reduces its chemotactic activity, either by binding to IP-10 or interfering with the binding of IP-10 to the CXCR3 receptor, still needs further study.

In conclusion, PrA mitigated recipient T cell migration and the infiltration of CXCR3^+^T cells into allografts via inhibition of IP-10/CXCR3 axis, which contributes to PrA immunosuppression. The results broaden our mechanistic understanding of PrA-altered immune responses and further support therapeutic potential of PrA for organ transplantation.
